# Creating Age-Friendly Communities: Housing and Technology

**DOI:** 10.3390/healthcare7040130

**Published:** 2019-11-03

**Authors:** Joost van Hoof, Hannah R. Marston, Katie R. Brittain, Helen R. Barrie

**Affiliations:** 1 Faculty of Social Work & Education, The Hague University of Applied Sciences, Johanna Westerdijkplein 75, 2521 EN Den Haag, The Netherlands; 2 Department of Spatial Economy, Faculty of Environmental Engineering and Geodesy, Wrocław University of Environmental and Life Sciences, ul. Grunwaldzka 55, 50-357 Wrocław, Poland; 3 Health & Wellbeing Priority Research Area, School of Health, Wellbeing & Social Care, The Open University, Milton Keynes, Buckinghamshire MK7 6HH, UK or; 4 Department of Nursing, Midwifery and Health, Northumbria University, Coach Lane Campus, Newcastle upon Tyne NE7 7XA, UK; katie.brittain@northumbria.ac.uk; 5 School of Social Sciences, The University of Adelaide, North Terrace, Adelaide, South Australia 5005, Australia; helen.barrie@adelaide.edu.au

Taking an international perspective of healthy ageing, people are living longer and are generally in better health than previous generations. Yet, given the rapidly increasing number of older adults, this demographic shift puts an increased level of stress on worldwide healthcare systems. The vast majority of older adults wish to age-in-place, to continue to live in their choice of residence for as long as they can. Yet, there is a small but significant percentage of older people who make use of long-term care services, including homecare, rehabilitation services, and social support. According to the Organisation for Economic Co-operation and Development (OECD) [1], cities in particular have large numbers of older citizens and are home to 43.2% of the older population. In order to stimulate and support urban ageing [2,3,4,5,6], cities can be improved to facilitate a more age-friendly environment. One way to support older people to live the life they wish to live is through the age-friendly cities initiative of the World Health Organization, a world-wide programme to improve cities to meet the needs of older citizens [7,8,9]. An age-friendly city offers a supportive environment that enables residents to grow older actively within their families, neighbourhoods, and civil society, and offers extensive opportunities for their participation in the community [10]. In addition, an age-friendly city optimises opportunities for health, participation, and security in order to enhance quality of life as people age [7]. 

According to the OECD [1], ageing societies pose diverse challenges, such as redesigning infrastructure and urban development patterns, social isolation, lack of accessibility and affordable housing. When referring to age-friendly cities, there are a lack of studies looking at the outcomes of age-friendly city approaches [3]. There are several questions that need to be addressed when building age-friendly places, inclusive environments and/or technologies. With advances in technology, engineering and design these domains offer a wide range of solutions to support daily function, activities and participation; facilitate the provision of healthcare, and offer means for leisure to older people. Too often, end-users of architectural and technological solutions are not consulted in the design processes and the implementation of the solutions in practice. Their inclusion in these processes is paramount to the success of the proposed and implemented solutions. Therefore, the purpose of this Special Issue is to present an overview of studies addressing recent advances in age-friendly cities in relation to housing (including urban planning) and technology, both in the broadest sense of the word. Apart from a focus on active and healthy ageing in cities, similar challenges can be found in rural areas too.

Creating age-friendly communities and environments are key in the 21st century, which cannot be ignored and must be continuously reviewed and refined to ensure respective environments—be-it housing of all environmental types encountered in one’s housing career (for instance, own home, nursing home, assisted living [11]), neighbourhoods and other public spaces; or the technologies that enable and assist with living independently—are up-to-date and meet the needs of the respective residents, carers and visitors. At the turn of the 21st century, citizens, residents, businesses, organisations, educationalists, health practitioners and policy makers were actors in society; be-it as spectators, observers, developers, entrepreneurs, care staff or other, engaged in the design, development, implementation and refining of information communication technologies and peripheral devices and software. The first two decades of the new millennium have been phenomenal from the standpoint of technology integration within the day-to-day lives of citizens. 

The WHO framework of age-friendly cities and communities [7] outlines very clearly the eight domains that make-up this framework and to date there has been a growth in research and collaboration between academe, policy makers, stakeholders and citizens on an international scale. The model itself can be extended to include the implementation of technology in the daily lives of older people [12,13]. Gerontechnology aims at good health, full social participation and independent living up to an advanced age, and to understand through research, development, design of products and services the means of increasing quality of life [14]. Both older people and carers may be amongst the end-users of such technological solutions [15]. Many contributions to this special issue deal with technology for older people.

This special issue of *Healthcare* on “Creating Age-Friendly Communities: Housing and Technology” is timely, comprising twelve papers [16,17,18,19,20,21,22,23,24,25,26,27] that traverse and intersect across the fields of gerontology, health and social care, social sciences and gerontechnology.

Statistics show age-related, long-term conditions such as dementia are a primary focus and concern for Western developed countries. There are currently over 47 million people worldwide living with dementia and this number is projected to increase to 75 million by 2030, and 135 million by 2050 [28]. Thus, the accepted papers surrounding dementia have focused on the perspectives of healthcare professionals in conjunction with meaningful activities for individuals diagnosed with dementia residing in a nursing home environment [16,25], and installing and using aids and adaptations within the home to create a physical environment that is more dementia-friendly [19]. Furthermore, exploring and understanding technology use associated with care of individuals living with dementia in the community from the perspective of stakeholders [23] is equally important as a systematic review to ascertain the current landscape and offer readers the ability to see what areas need greater improvement and expansion.

Five papers [16,17,19,20,23] take the standpoint of technology use and deployment within various social contexts as a means of contributing to the national and international discussions and debates surrounding the age-friendly landscape. Across these accepted papers, we have demonstrated the growth in multi-and-cross disciplinary research, which intersects at various disciplines across academia but also at policy levels associated with national [29] and devolved governments [30,31]. Huisman and Kort [17] present the research study “Two-Year Use of Care Robot Zora in Dutch Nursing Homes: An Evaluation Study” focusing on the deployment of Zora the robot into fourteen residential care environments, and reported on the barriers experienced by healthcare professionals, which included software failures and the start-up time, whilst the enablers of using such technology were seen positively by service users, adding additional value to the work given by the healthcare professionals. Marston and Samuels [18] take a position standpoint, focusing on the use of virtual assistants within the home and the benefits such virtual assistants can have on dependent children and carers in later life. The respective authors extend their position by opening up the discussion surrounding the age-friendly environment, and the need for greater intergenerational focus, by proposing a series of recommendations and future work to expand the fields of gerontology, social science and gerontechnology. Chadborn and colleagues [20] discuss the positive and negative perceptions and attitudes towards digital health technologies. Conducting a citizen jury approach, Chadborn and colleagues were able to execute a deliberative inquiry into such benefits as well as risks surrounding smart health technologies and systems. Findings from this empirical research ascertained respective participants felt their views were largely ignored by organisations who were responsible to implementing such systems. Wang and colleagues [21] explored the perspectives of ambient-assisted living and artificial intelligence technologies by older adults taking a user-centered design approach as a preliminary stage to participating in a co-design process. A survey was deployed to collect privacy perspectives, followed by two 90-minute focus groups with 31 community residents. Findings highlighted low digital literacy, which included unfamiliar terminology coupled with physical challenges, making technology adoption difficult. However, positive facilitators showed participants eagerness to learn, be part of co-production, and to understand their data. Furthermore, participants showed an interest in having their voice heard relating to the design of specific technologies to successfully age-in-place. Marston and colleagues [24] reported the overall findings from the Technology In Later Life (TILL) study, which was conducted across four sites located in two countries (United Kingdom and Canada). The sites were either rural or urban, and the study aimed to understand the role in which technology impacts the lives of adults aged 65+ years. Recommendations were proposed as a way of improving the lives and social connectedness of older citizens, while for many participants living in rural locations, the use of technology such as Skype was greater for maintaining a connection with children and grandchildren living across the country or on a different continent. Lee and colleagues [22] explore in their body of work “Living Alone Among Older Adults in Canada and the U.S.”, Canadian and American data sets to understand the living arrangements of older adults and how one’s living arrangement can affect wellbeing, whilst informing respective housing needs. In a second part of the data analysis, they explore the various factors of immigrants who live alone. Based on these findings, Lee and colleagues propose a greater need for innovative design and technology relating to age-friendly housing, in particular for older adults who live alone, and state that attention is needed when designing age-friendly housing for immigrants who may have different needs, requirements and cultural preferences. Barrie and colleagues [26] explore and discuss external environments, relating to the impact of good design and accessibility to mobility, independence, quality of life, and the ability of older adults to age-in-place. This paper deployed a citizen science approach to data collection, using an audit tool on smartphones to assess neighbourhood public green spaces. Citizen science data included photographs, geo-coded location, survey data, and qualitative-based comments. This submission uses an existing and popular methodological approach found in natural sciences, but less prevalent in the social sciences to understand, from the older residents’ perspective, what makes a good public green space for ageing well.

Finally, the last paper in this special issue is a scoping review by Marston and colleagues [27], exploring contemporary literature surrounding mobile electrocardiogram devices available on the market to consumers and offer healthcare professionals the opportunity to remote monitor patient’s health concerns of arrhythmia and palpitations. The “Mobile Self-monitoring ECG Devices to Diagnose Arrhythmia (AR) that coincide with Palpitations: A Scoping Review” offers an insight into how specific technology can be deployed and used by specific professions and citizens. The respective authors propose future work and recommendations to extend this work and include the need for work to be conducted and evaluated in low, middle, income countries, and different geographic locations, and to understand the adoption and adherence of this type of technology from both the patient and provider perspective.

Age-friendly initiatives focusing on how communities can support older people to age-in-place have gathered momentum in academic and policy circles [32]. However, although there is a growing evidence base that demonstrates the positive impact that age-friendly environments can have on older people, many environments still remain a challenge for older people. Technologies aimed at supporting older people to age-in-place have been proposed as one solution to overcoming these environmental challenges. We know that older people are situated within a complex array of ‘material/physical, social and psychological relations and affects’ and that there is a need to go beyond just focusing on technological innovation as a solution [33]. The papers in this collection clearly highlight the complexities around what constitutes an age-friendly environment, from the perspective of older people, people with dementia, formal and informal carers, and health and social care professionals. Living arrangements and environments in the broadest sense are explored in this volume of work as to how they impact on ageing. Supporting nursing home residents in meaningful activities [25], the impact of living alone [22], the use of aids and adaptations to support well-being [19] and the importance of public green space [16,26] all contribute to a discussion as to how to create an age-friendly environment. Importantly, a number of included papers highlight the ‘expert’ role older people have in their daily experience of the environments in which they live [26] and research in this area needs to move away from technological innovations that are not rooted in this expertise. The implementation of citizen science methodologies follows that of co-design and co-research [34,35,36], a movement that may lead age-friendly cities to become age-inclusive cities. When creating age-friendly communities, including housing and technology, the voices of people of all age groups matter and should be heard. The scholarly work included in this special issue may help societies move forward in the quest to become truly age-friendly.

## References

[1] OECD (2015). Ageing in Cities.

[2] Van Hoof J., Kazak J.K. (2018). Urban ageing. Indoor Built Environ..

[3] Van Hoof J., Kazak J.K., Perek-Białas J.M., Peek S.T.M. (2018). The challenges of urban ageing: Making cities age-friendly in Europe. Int. J. Environ. Res. Public Health.

[4] Buffel T., Phillipson C., Skinner M.W., Andrews G.J., Cutchin M.P. (2018). Urban ageing: New agendas for geographical gerontology. Geographical Gerontology: Perspectives, Concepts, Approaches.

[5] Buffel T., Phillipson C. (2018). A manifesto for the age-friendly movement: Developing a new urban agenda. J. Aging Soc. Policy.

[6] Kazak J., van Hoof J., Świąder M., Szewrański S. (2017). Real estate for the ageing society—The perspective of a new market. Real Estate Manag. Valuat..

[7] World Health Organization (2007). Global Age-Friendly Cities: A Guide.

[8] Plouffe L., Kalache A. (2010). Towards global age-friendly cities: Determining urban features that promote active aging. J. Urban Health.

[9] Buffel T., Phillipson C. (2016). Can global cities be ‘age-friendly cities’? Urban development and ageing populations. Cities.

[10] Caro F.G., Fitzgerald K.G. (2016). International Perspectives on Age-Friendly Cities.

[11] Van Hoof J., Kort H.S.M., van Waarde H. (2009). Housing and care for older adults with dementia. A European perspective. J. Hous. Built. Environ..

[12] Marston H.R., van Hoof J. (2019). Who doesn’t think about technology when designing urban environments for older people? A case study approach to a proposed extension of the WHO’s age-friendly cities model. Int. J. Environ. Res. Public Health.

[13] Van Hoof J., Dikken J., Buttiġieġ S.C., van den Hoven R.F.M., Kroon E., Marston H.R. (2019). Age-friendly cities in the Netherlands: An explorative study of facilitators and hindrances in the built environment and ageism in design. Indoor Built. Environ..

[14] Van Bronswijk J.E.M.H., Bouma H., Fozard J.L. (2002). Technology for quality of life: An enriched taxonomy. Gerontechnology.

[15] Oude Weernink C.E., Felix E., Verkuijlen P.J.E.M., Dierick-van Daele A.T.M., Kazak J.K., van Hoof J. (2018). Real-time location systems in nursing homes: State of the art and future applications. J. Enabling Technol..

[16] De Boer B., Beerens H.C., Katterbach M., Viduka M., Willemse B.M., Verbeek H. (2018). The physical environment of nursing homes for people with dementia: Traditional nursing homes, small-scale living facilities, and green care farms. Healthcare.

[17] Huisman C., Kort H. (2019). Two-year use of care robot Zora in Dutch nursing homes: An evaluation study. Healthcare.

[18] Marston H.R., Samuels J. (2019). A review of age-friendly virtual assistive technologies and their effect on daily living for carers and dependent adults. Healthcare.

[19] Evans S., Waller S., Bray J., Atkinson T. (2019). Making homes more dementia-friendly through the use of aids and adaptations. Healthcare.

[20] Chadborn N.H., Blair K., Creswick H., Hughes N., Dowthwaite L., Adenekan O., Pérez Vallejos E. (2019). Citizens’ juries: When older adults deliberate on the benefits and risks of smart health and smart homes. Healthcare.

[21] Wang S., Bolling K., Mao W., Reichstadt J., Jeste D., Kim H.-C., Nebeker C. (2019). Technology to support aging in place: Older adults’ perspectives. Healthcare.

[22] Lee S.M., Edmonston B. (2019). Living alone among older adults in Canada and the U.S.. Healthcare.

[23] Van Boekel L.C., Wouters E.J.M., Grimberg B.M., van der Meer N.J.M., Luijkx K.G. (2019). Perspectives of stakeholders on technology use in the care for community-living older adults with dementia: A systematic literature review. Healthcare.

[24] Marston H.R., Genoe R., Freeman S., Kulczycki C., Musselwhite C. (2019). Older adults’ perceptions of ICT: Main findings from the Technology in Later Life (TILL) study. Healthcare.

[25] Groenendaal M., Loor A., Trouw M., Achterberg W.P., Caljouw M.A.A. (2019). Perspectives of healthcare professionals on meaningful activities for persons with dementia in transition from home to a nursing home: An explorative study. Healthcare.

[26] Barrie H., Soebarto V., Lange J., Mc Corry-Breen F., Walker L. (2019). Using citizen science to explore neighbourhood influences on ageing well: Pilot project. Healthcare.

[27] Marston H.R., Hadley R., Banks D., Del Carmen Miranda Duro M. (2019). Mobile self-monitoring ECG devices to diagnose arrhythmia that coincide with palpitations: A scoping review. Healthcare.

[28] Alzheimer’s Disease International (2013). Policy Brief for G8 Heads of Government. The Global Impact of Dementia 2013-2050. London: Alzheimer’s Disease International. https://www.alz.co.uk/research/GlobalImpactDementia2013.pdf.

[29] Marston H.R., Musselwhite C. (2019). Ageing: Science, Technology & Healthy Living; Written evidence (INQ0010), Questions 5–8. Science and Technology Committee (Lords), UK. http://data.parliament.uk/writtenevidence/committeeevidence.svc/evidencedocument/science-and-technology-committee-lords/ageing-science-technology-and-healthy-living/written/104933.html.

[30] Waights V., Bamidis P., Almeida R. (2018). Technologies for Care—The Imperative for Upskilling Carers. Knowledge Exchange Seminar Series 2017–18.

[31] Marston H.R., Freeman S., Genoe R., Kulcyzki C., Musselwhite C. (2018). The Cohesiveness of Technology in Later Life: Findings from the Technology. Later Life (TILL) Project Knowledge Exchange Seminar Series 2017–2018.

[32] Buffel T., Handler S., Phillipson C. (2018). Age-Friendly Cities and Communities: A Global Perspective.

[33] Buse C., Martin D., Nettleton S. (2018). Conceptualising ‘materialities of care’: Making visible mundane material culture in health and social care contexts. Sociol. Health Illn..

[34] Kort H.S.M., Steunenberg B., van Hoof J. (2019). Methods for involving people living with dementia and their informal carers as co-developers of technological solutions. Dement. Geriatr. Cogn. Disord..

[35] Van Hoof J., Verbeek H., Janssen B.M., Eijkelenboom A., Molony S.L., Felix E., Nieboer K.A., Zwerts-Verhelst E.L.M., Sijstermans J.J.W.M., Wouters E.J.M. (2016). A three perspective study of the sense of home of nursing home residents: The views of residents, care professionals and relatives. BMC Geriatr..

[36] Van Hoof J., Verboor J., Oude Weernink C.E., Sponselee A.A.G., Sturm J.A., Kazak J.K., Govers G.M.J., van Zaalen Y. (2018). Real-time location systems for asset management in nursing homes: An explorative study of ethical aspects. Information.

